# Functional Inactivation of EBV-Specific T-Lymphocytes in Nasopharyngeal Carcinoma: Implications for Tumor Immunotherapy

**DOI:** 10.1371/journal.pone.0001122

**Published:** 2007-11-07

**Authors:** Jiang Li, Xue-hui Zeng, Hao-yuan Mo, Ulrika Rolén, Yan-fang Gao, Xiao-shi Zhang, Qiu-yan Chen, Li Zhang, Mu-sheng Zeng, Man-zhi Li, Wen-lin Huang, Xiao-ning Wang, Yi-Xin Zeng, Maria G. Masucci

**Affiliations:** 1 State Key Laboratory of Oncology in Southern China, Cancer Center, Sun Yat-sen University, Guangzhou, China; 2 Department of Experimental Research, Cancer Center, Sun Yat-sen University, Guangzhou, China; 3 Department of Biotherapy, Cancer Center, Sun Yat-sen University, Guangzhou, China; 4 Department of Nasopharyngeal Carcinoma, Cancer Center, Sun Yat-sen University, Guangzhou, China; 5 Department of Radiotherapy, Cancer Center, Sun Yat-sen University, Guangzhou, China; 6 Institute of Molecular Immunology, School of Biotechnology, Technology University of Southern China, Guangzhou, China; 7 Department of Cell and Molecular Biology, Karolinska Institutet, Stockholm, Sweden; New York University School of Medicine, United States of America

## Abstract

Nasopharyngeal carcinoma (NPC) is an Epstein-Barr virus (EBV) associated malignancy with high prevalence in Southern Chinese. In order to assess whether defects of EBV-specific immunity may contribute to the tumor, the phenotype and function of circulating T-cells and tumor infiltrating lymphocytes (TILs) were investigated in untreated NPC patients. Circulating naïve CD3^+^CD45RA^+^ and CD4^+^CD25^−^ cells were decreased, while activated CD4^+^CD25^+^ T-cells and CD3^−^CD16^+^ NK-cells were increased in patients compared to healthy donors. The frequency of T-cells recognizing seven HLA-A2 restricted epitopes in LMP1 and LMP2 was lower in the patients and remained low after stimulation with autologous EBV-carrying cells. TILs expanded in low doses of IL-2 exhibited an increase of CD3^+^CD4^+^, CD3^+^CD45RO^+^ and CD4^+^CD25^+^ cells and 2 to 5 fold higher frequency of LMP1 and LMP2 tetramer positive cells compared to peripheral blood. EBV-specific cytotoxicity could be reactivated from the blood of most patients, whereas the TILs lacked cytotoxic activity and failed to produce IFNγ upon specific stimulation. Thus, EBV-specific rejection responses appear to be functionally inactivated at the tumor site in NPC.

## Introduction

Nasopharyngeal carcinoma (NPC) is an epithelial neoplasm that occurs at low frequency worldwide (<1 case per 10^5^ individuals/year), but at a more than 100 fold higher frequency in South-East Asia, North Africa and Alaska (30–80 cases per 10^5^ individual/year in Southern China) (reviewed in [1]. NPC can be categorized into three histological types: Type I, keratinizing squamous-cell carcinomas (SSC); Type II, non-keratinizing squamous carcinoma; Type III, undifferentiated carcinoma, [2]. In North America 25% of NPCs are histological Type I, 12% Type II, and 63% Type III, while in Southern China the distribution is 2%, 3%, and 95%, respectively. The pathogenesis of NPC is complex and both genetic and environmental factors are believed to play important roles [1], [3]. In addition, the regular detection of Epstein Barr virus (EBV) in the malignant cells of virtually all cases of NPC Type II and Type III [4] suggests that the virus is a determinant factor in oncogenesis.

EBV is a γ-herpes virus that causes asymptomatic life-long persistent infections in >90% of the adults population worldwide and is associated with a variety of malignancies of lymphoid, epithelial and mesenchimal cell origin including, in addition to NPC, EBV Burkitt's lymphoma (BL), NK/T cells lymphoma, Hodgkin lymphoma (HD), immunoblastic lymphoma and lejomyosarcoma arising in immunosuppressed and HIV patients and some histological types of gastric cancer (reviewed in [3], [5]. The malignant cells of these tumors sustain non productive EBV infections characterized by the expression of a restricted set of viral genes that, in different combinations, define three types of viral latency. In latency I, seen in BL, only the EBERs and BARTs transcripts and the EBV nuclear antigen (EBNA)-1 are expressed. In latency II, seen in EBV positive HD and NPC, the latent membrane proteins (LMP)-1 and -2 are expressed together with the latency I products, while six EBV nuclear antigens (EBNA1-6), LMP1 and -2 are expressed in latency III, seen in lymphoproliferative disorders associated with immunosuppression. Type III latency is also expressed in EBV immortalized lymphoblastoid cell lines (LCLs) obtained by *in vitro* EBV infection of normal B-lymphocytes or by spontaneous outgrowth from the blood of virtually all EBV carriers.

Vigorous humoral and cellular immune responses control the proliferation of EBV infected cells in healthy virus carriers. Both non-specific, NK-cell mediated, and EBV-specific, T-cell mediated, responses were shown to play important roles during primary infection (reviewed in [6], while EBV-specific T cells appear to be critically involved in restraining the proliferation of EBV infected cells during life-long persistent infection [7], [8]. Recent data obtained by staining with EBV peptides loaded HLA tetramer have confirmed that T cells specific for EBV antigens expressed during latent and productive infection are maintained in the blood of healthy carriers at relatively high frequencies throughout life [9]. Direct evidence for the importance of these EBV-specific T cells in controlling the oncogenic capacity of the virus is provided by the occurrence of EBV associated immunoblastic lymphomas in the patients where their activity is impaired by congenital immunodiciency, immunosuppressive therapy or HIV infection [10]. These EBV associated lymphomas can be prevented or even cured by adoptive transfer of *in vitro* activated and expanded EBV-specific T cells [11], [12], [13], suggesting that reconstitution of EBV-specific immunity may be a useful strategy in the management of EBV associated malignancies.

While the contribution of immunosuppression to the pathogenesis of immunoblastic lymphoma is well established, the failure of EBV specific immunity in other EBV associated malignancies is not well understood since the affected patients are usually fairly immunocompetent. The capacity of the tumor cells to evade EBV specific immunity has been proposed to play a role in the pathogenesis of BLs since the tumor cells exhibit a non-immunogenic phenotype and their viral antigen expression is restricted to EBNA-1 that was shown to be a poor target for MHC class I restricted rejection responses [14], [15], [16]. However, evidence collected from both HD and NPC suggests that, in accordance with their expression of immunogenic viral antigens, the malignant cells of these tumors are efficiently recognized by EBV specific T cell [17]. A selective impairment EBV specific responses was demonstrated at the tumor site in EBV positive HD [18], suggesting that local modulation of immune responses may promote the escape of tumor cells. It is not known whether a site-specific suppression of rejection responses occurs in other EBV associated malignancies.

In this study we have addressed the status of EBV specific immunity in NPC patients by investigating the phenotype, frequency of EBV specific T cells and capacity to mount cytotoxic responses upon stimulation with autologous EBV carrying cells in the blood ofnewly diagnosed NPC patients and in tumor infiltrating lymphocytes (TILs) from a subset of the patients. We show that efficient EBV-specific CTL responses can be reactivated in blood lymphocytes from the majority of NPC patients, in spite of a significant decrease in the number of circulating T lymphocytes and in the frequency of cells recognizing LMP1 and LMP2 epitopes. LMP1 and LMP2 epitopes-specific T cells were enriched in TILs expanded in IL-2 but t were not cytotoxic and failed to produce IFN-γ upon specific stimulation. Thus, EBV-specific rejection responses appear to be selectively inhibited at the tumor site in NPC.

## Results

### EBV antibody titers, virus load and serum levels of cytokines

**Figure 1:**
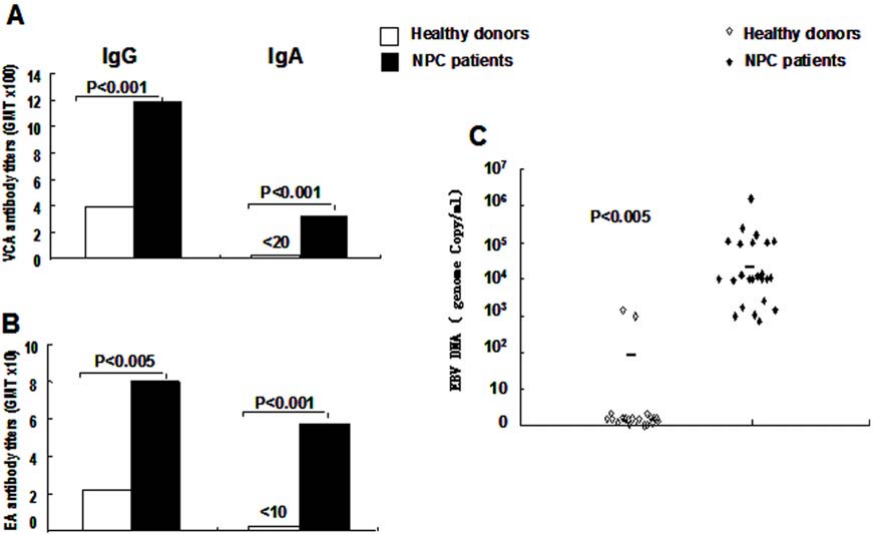
EBV VCA and EA antibody titers and EBV DNA in the serum of NPC patients and healthy EBV carriers. Antibody titers to EBV VCA IgG and IgA (A) and EA IgG and IgA (B) in the serum of healthy donors (n = 20) and NPC patients (n = 40); EBV DNA load (C) in healthy donors (n = 20) and NPC patients (n = 24); ns =  non significant.

Heparinized venous blood and tumor biopsies were collected from 40 untreated NPC patients at the time of first remittal to the Nasopharyngeal Carcinoma Unit of the Cancer Center of Sun-yat Sen University, and blood was collected from 20 healthy volunteers. The clinical diagnosis of NPC was confirmed by histopathology in all cases and the expression of EBER1 was confirmed by ISH in 30 biopsies (Table 1). Previous EBV infection was confirmed by the presence of IgG-VCA antibodies in all patients and controls (Figure 1). IgG titers to VCA and EA were significantly higher in patients compared to controls (P<0.001 and P<0.005 respectively, Figure 1A and 1B) while IgA antibodies to VCA and EA were detected in 88% and 70% of the patients, respectively, but not in healthy EBV carriers (P<0.001).

**Table 1:** Samples information.

	Gender/age	HLA type	NPC type^b^	WHO classification^c^	Clinical Stage^d^	EBV status
P1	M/30	A02,11; B4001,51	III	T4N1M0	IVa	+
P2	M/32	A02,33; B1501,35	III	T4N2M0	IVa	+
P3	M/50	A02; B13,38	III	T2N1M0	II	+
P4	F/58	A02,11; B13,46	III	T2N2M0	III	+
P5	F/40	A11,33; B44,46	III	T4N0M0	IVa	+
P6	M/35	A02,33; B65,58	I	T4N1M0	IVa	+
P7	M/30	A02; B46	III	T3N2M0	III	+
P8	M/33	A02,11; B60,75	III	T4N2M0	IVa	+
P9	M/60	A02; B13,38	III	T4N3M0	IVa	+
P10	M/33	A02; B46,58	III	T4N1M0	IVa	+
P11	M/35	A02,24;B1501	III	T3N2M0	IVa	+
P12	F/53	A11,26; B13,44	I	T4N2M0	IVa	+
P13	M/53	A02,33; B46,57	III	T3N2M0	III	+
P14	F/38	nd^e^	III	T4N1M0	IVa	+
P15	M/41	nd	III	T3N1M0	III	+
P16	M/43	nd	III	T3N2M0	III	+
P17	F/51	A02,24; B46,58	III	T3N2M0	III	+
P18	F/38	nd	III	T3N2M0	III	nd
P19	M/57	A11,33; B15(62);58	III	T3N2M0	III	+
P20	F/50	A1133; B58,62(15)	III	T3N2M0	III	+
P21	M/47	A02,02; B46,58	III	T3N1M0	III	+
P22	M/33	nd	III	T2N2M0	III	+
P23	M/35	nd	III	T2N1M0	II	nd
P24	M/36	A02,11; B46,1502	III	T2N0M0	II	+
P25	M/47	A01,11; B40(60),54	III	T2N1M0	II	+
P26	M/46	A24;B40(60)	III	T3N2M0	III	nd
P27	F/72	nd	III	T3N2M0	IVa	+
P28	M/46	nd	III	T3N2M0	III	nd
P29	M/34	nd	III	T2N1M1	IVb	nd
P30	M/76	A02,11; B13,46	III	T3N1M0	III	nd
P31	M/38	A02,02; B38,44	III	T4N0M0	IVa	+
P32	M/57	nd	III	T4N0M0	IVa	nd
P33	M/51	A11,33; B38,58	III	T2N2M0	III	+
P34	M/47	A02,24; B38,40(60)	III	T3N2M0	III	nd
P35	M/53	A02,24;B15,46	III	T2N2M0	III	+
P36	M/33	A02,11; B46	III	T2N2M0	II	+
P37	M/78	A02,33;B46,58	III	T2N3M0	III	+
P38	M/30	A02,11; B15(75),38	III	T4N4M4	IVb	+
P39	M/56	A02,24; B40(61),54	III	T2N3M0	IVa	nd
P40	M/30	A02,11;B15(76),40(60)	I	T2N2M0	III	nd
N1	M/19	nd	-	-	-	−
N2	F/20	nd	-	-	-	−
N3	M/22	nd	-	-	-	−
N4	M/19	nd	-	-	-	−
N5	F/21	A02,11; B13,75(15)	-	-	-	−
N6	M/22	A02,11;B46	-			
N7	M/23	A02,33; B46,58	-	-	-	−
N8	M/24	A26,30;B13,40(61)	-	-	-	−
N9	M/22	A11; B39,53	-	-	-	−
N10	M/22	A33; B58	-	-	-	−
N11	F/22	nd	-	-	-	−
N12	F/21	A01,11; B15(63),15(75)	-	-	-	−
N13	M/23	A02,11;B13,51	-	-	-	−
N14	F/24	A02,33; B46,58	-	-	-	−
N15	F/23	A02;B13,52	-	-	-	−
N16	F/23	A02;B15,40	-	-	-	−
N17	M/25	A11,33; B40(60),58	-	-	-	−
N18	M/53	A11,24;B4001	-	-	-	−
N19	M/57	A02,11;B46,56	-	-	-	−
N20	M/35	A02,11;N13,46	-	-	-	−

a P = NPC patient; N = healthy donors.

b Histological type: I = differentiated keratinizing squamous cell carcinoma (SCC); II = differentiated non-keratinizing carcinoma; III = undifferentiated carcinoma (UCNT).

c TNM stage according to WHO UICC-AJCC 1997 standards (International Union Against Cancer/American Joint Committee on Cancer).

d Clinical stage: Stage I = T1N0M0; Stage II = T2N0-1M0; T0-1N1M0; Stage III = T3N0-2M0 or T0-3N2M0; Stage IVa = T4N0-3M0; T0-4N3M0 or Stage IVb = all T and all N plus M1.

e nd = not done.

**Figure 2:**
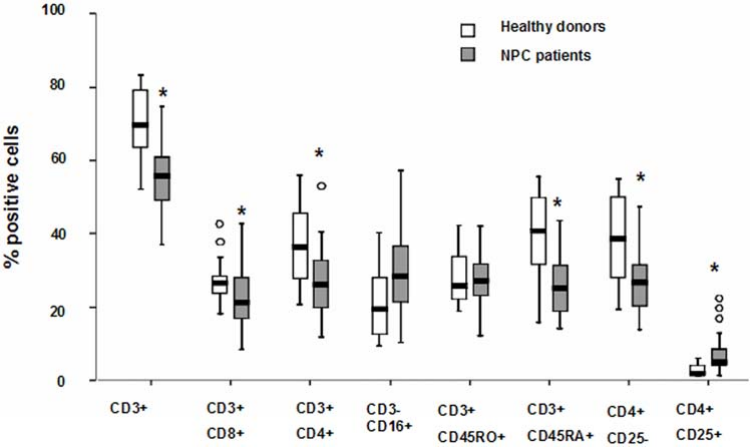
Immunophenotype of PBMCs from healthy donors and NPC patients. Surface markers analysis was performed by directly immunofluorescence labeling and FACS analysis in PBMCs from healthy donors (n = 20) and NPC patients (n = 40). * P<0.01

The presence of EBV DNA was investigated by real time quantitative PCR in the serum of 24 NPC patients and 20 controls (Figure 2B). Between 10^3^ to >10^6^ genome copy/ml serum were detected in all patients, while less than 10^4^ copies/ml were detected only in 2 out of 20 healthy donors (P<0.01).

The serum levels of Th1 (IFN-γ, IL-2, TNF-α) and Th2 (IL-4, IL-6, IL-10) cytokines were analyzed in 27 patients and 19 controls. The serum levels of all cytokines were below the sensitivity of the CBA in all but one healthy control where small amounts of IL-2 were detected, while IFN-γ (3.2±3.0 pg/ml), IL-6 (2.8±3.0 pg/ml) and IL-10 (1.5±1.3 pg/ml) were detected in the serum of the majority of the patients (Table 2). Low levels of TNFα, IL-2 and IL-4 were also detected in few patients.

**Table 2:** Serum levels of Th1 and Th2 cytokines.

Cytokine	Healthy donors		NPC Patients	
	Positive/Total (%)	Mean pg/ml	Positive/Total (%)	Mean pg/ml
**IFN-γ**	0/19 (0)	0	19/27 (70)	3.2*
**TNF-α**	0/19 (0)	0	9/27 (33)	0.6
**IL-2**	1/19 (5)	0.2	4/27 (15)	0.4
**IL-4**	0/19 (0)	0	3/27 (11)	0.2
**IL-6**	0/19 (0)	0	21/27 (78)	2.8*
**IL-10**	0/19 (0)	0	20/27 (74)	1.5*

* Significant different from healthy donors (P<0.0001).

### T cell phenotype and frequency of EBV specific T cells in PBMCs

To evaluate the distribution of lymphocyte subsets, the expression of surface markers specific for T cells: CD3, CD4, CD8; NK cells: CD16; naïve, memory and activated T cells: CD45RO, CD45RA and CD25, was detected by immunoflourescence staining and FACS analysis in PBMCs from all patients and controls (Figure 2). The percentage of CD3^+^, CD3^+^CD8^+^ and CD3^+^CD4^+^ T cells were significantly decreased in the patients (56±12.5, 21±8.8 and 26±9.5 compared to 68±9.3, 27±6.5 and 34±10.7, respectively), and this was companied by a relative increase of CD3^−^CD16^+^ NK cells (28.1±11.1 compared to 22±9.5). The patients also showed a significant decrease in CD3^+^CD45RA^+^ and CD4^+^CD25 naïve T cells (24±9.0 and (25.4±8.5 compared to 39±12.2 and 37.8±11.5, respectively) while there was no difference in the percentage of T cells expressing a CD45RO^+^CD3^+^, memory phenotype (26.1±7.6 and 28.1±6.8, respectively). A significantly higher percentage of CD4^+^CD25^+^ activated T lymphocytes was detected in the patients compared to healthy donors (7±4.9 and 3±1.6, respectively)

**Figure 3:**
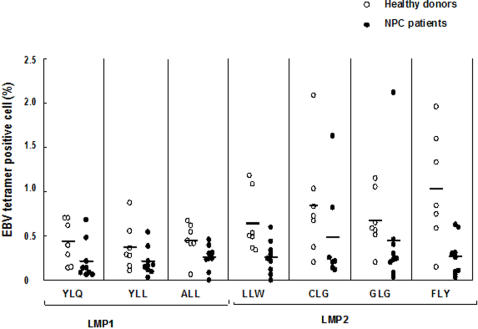
Frequency of LMP1 and LMP2 epitope- specific T cells in PBMCs from healthy donors and NPC patients. The frequency of T cells specific for HLA A2 restricted on LMP1 and LMP2 was detected by EBV tetramer staining and FACS analysis in freshly isolated PBMCs from healthy donors (n = 7) and NPC patients (n = 9). The number of tetramer positive cells in 100 lymphocytes are shown in the figure.

The presence of CD3^+^CD8^high^ T cell able to recognize epitopes in LMP1 (YLQ, YLL, ALL) and LMP2 (LLW, CLG, GLG, FLY) was investigated by tetramer staining in PBMCs from 9 HLA-A2 positive patients and 7 controls (Figure 3 and Supplementary Information Figure S2). The percentage of tetramer positive cells was generally lower in the patients and the differences were statistically significant as assayed by Mann-Whitney U test (P<0.05) for 2 out of 3 LMP1 epitopes (YLQ: 0.22 and 0.34, ALL: 0.25 and 0.64, respectively), and 3 out of 4 LMP2 epitopes (LLW: 0.26 and 0.64, GLG: 0.45 and 0.67, FLY: 0.27 and 1.03, respectively). In line within the observation that LMP2 contains several immunodominant HLA-A2 restricted epitopes [19], the PBMCs of healthy donors showed higher frequencies of cells stained with LMP2 compared with LMP1 tetramers (P = 0.003, Mann-Whitney U test), but this difference were not seen in PBMCs from NPC patient.

### Reactivation of EBV-specific CTLs and LMP1 and LMP2 tetramer positive cells

**Figure 4:**
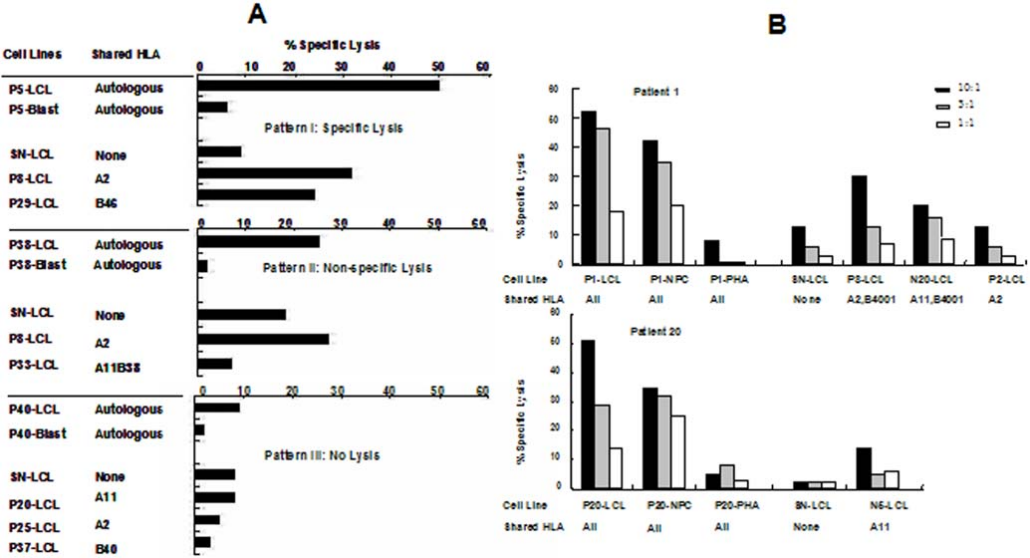
Cytotoxic activity of auto-LCL activated cultures from PBMCs of NPC patients. Polyclonal CTL cultures were test for cytotoxic activity against a panel of targets including the autologous EBV transformed LCL, PHA blasts, allogenic HLA class I matched or mismatched LCL. A. Representative experiments illustrating three pattern of cytotoxic activity. Pattern I: Lysis of the auto-LCL ≥25% and HLA class I mismatched LCL ≤10%; Pattern II: lysis to both HLA class I matched and mismatched LCL; Pattern III: less than 10% lysis against autologous or allogenic EBV positive or negative targets B. EBV-specific CTL cultures from NPC patients lysed freshly isolated autologous NPC tumor cells. Representative ^51^Cr release assays performed with CTL cultures from two NPC patients are shown in the figure.

In order to assess the presence of circulating EBV specific CTLs precursors, PBMCs from 15 NPC patients and 8 controls were stimulated with the autologous LCL and the cytotoxic activity of the cultures was tested against a panel of EBV positive and negative targets. Efficient expansion of CD3^+^CD8^+^ T cells expressing CD45RO^+^ was achieved in all cases (not shown). Three patterns of cytotoxicity were recognized based on differential killing of EBV^+^ or EBV^−^ targets (Figure 4A and Table 3): Pattern I: EBV specific cytotoxicity, characterized by efficient lysis (≥25% at 10∶1 R∶S ratio) of the autologous and allogenic HLA class I matched LCLs and no lysis of autologous PHA blast and HLA class I mismatched targets, Pattern II: LAK-type cytotoxicity, characterized by equal lysis of HLA class I matched and mismatched LCLs and Pattern III: no cytotoxicity, where the specific lysis against all targets was ≤10% at 10∶1 R∶S ratio. EBV specific cytotoxicity could be reactivated in 8 out 10 controls (80%) and in a slightly lower proportion of patients (10 out of 15, 66%) while LAK-type response was obtained in 2 donors (20%) and 3 patients (20%) and cytotoxicity was not detected in the cultures from 2 patients (13%) (Table 3), The lytic capacity of auto-LCL stimulated cultures was also supported by the presence of CD3^+^CD107a^+^ T cells in cultures incubated for 4 hrs with different targets cells or peptides (Supplementary Information Table S1). A Pattern I cytotoxicity was associated with efficient lysis of autologous primary tumor cells in the CTL cultures derived from 2 NPC patients (Figure 4B).

**Table 3:** Patterns of cytotoxicity induced by stimulation of PBMCs with the autologous LCL.

	Autologous LCL^a^	Autologous PHA blast	HLA Mismatched LCL	Pattern^b^
**P1**	+++	−	−	I
**P5**	+++	−	−	I
**P13**	++	−	+	II
**P17**	++	−	−	I
**P19**	++	−	−	I
**P20**	+++	−	−	I
**P30**	++	−	−	I
**P31**	+	−	−	II
**P32**	++	−	−	I
**P33**	++	−	+	II
**P34**	+	−	−	II
**P35**	++	−	−	I
**P36**	+	−	−	II
**P37**	+	−	+	II
**P40**	−	−	−	III
**N5**	++	−	−	I
**N6**	++	−	−	I
**N14**	++	−	−	I
**N16**	++	−	−	I
**N15**	++	−	−	I
**N12**	++	−	−	I
**N18**	+	−	++	II
**N20**	+	−	+	II

a − = Specific lysis <10%; + = Specific lysis 10–25%; ++ = Specific lysis 25–50%; +++ = Specific lysis >50%.

b Score: Pattern I = Specific lysis; Pattern II = non-specific lysis; Pattern III = no lysis.

**Figure 5:**
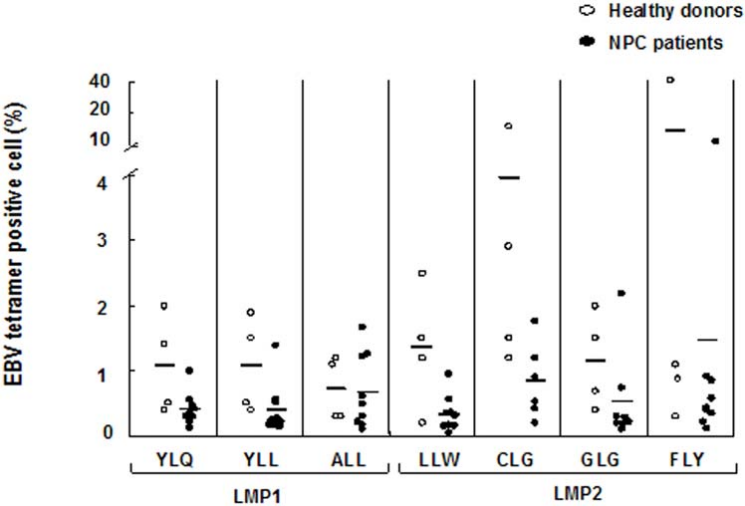
Frequency of LMP1 and LMP2 epitope- specific T cells in auto-LCL stimulated cultures from healthy donors and NPC patients. The frequency of T cells specific for HLA A2 restricted epitopes in LMP1 and LMP2 was detected by EBV tetramer staining and FACS analysis in auto-LCL stimulated cultures from healthy donors (n = 4) and NPC patients (n = 9).

Tetramer staining was performed to investigate the frequency of T cells specific for LMP1 and LMP2 epitopes in CTL cultures from 9 HLA-A2 positive patients and 4 controls (Figure 5, Table 4). As observed in PBMCs, the overall frequency of T cells stained with the LMP1 and LMP2 tetramers remained lower in the patients compared to controls and the differences were statistically significant as assayed by Mann-Whitney U test (P<0.05) for 1 out of 3 LMP1 epitopes (YLL 0.40 and 1.08, respectively), and 2 out of 4 LMP2 epitopes (LLW 0.33 and 1.41, CLG 0.83 and 3.97, respectively) although the frequency of tetramer positive cells was increased following LCL stimulation in all cases (Figure 5). The efficiency of the response against the various epitopes appeared to be somewhat different. Thus, while the CLG and FLY epitopes in LMP2 were clearly preferred in the healthy donors, T cells recognizing the ALL and YLL epitopes in LMP1 and CLG epitope in LMP2 were preferentially expanded in the patients and the response to ALL and YLL was stronger in NPC patients compared to healthy EBV carriers (Table 4, and Supplementary Information Figures S2 and S3, Tables S1 and S2).

**Table 4:** A activation of EBV epitope specific T cells in auto-LCL stimulated PBMCs (ALSC) and TILs.

	Increase of epitope specific T cells^a^						
	YLQ	YLL	ALL	LLW	CLG	GLG	FLY
**Healthy ALSC/PBMC (n = 3)**	3.9	3.8	2.5	4	11.7	3.2	15.7
**Patients ALSC/PBMC (n = 4)**	1.4	4.2	3.5	1.6	4.5	1.3	2.5
**Patients TIL/PBMC (n = 3)**	4.2	3.8	1.6	2.6	3	4.7	2.6

a Fold increased was calculated as mean % tetramer positive cells in ALSC or TILs/ mean % tetramer positive cells from PBMCs. Frequency of T cells recognizing LMP1 or LMP2 epitopes was determined by tetramer staining in paired samples derived form the donors.

### Phenotypic and functional characterization of TILs

**Figure 6:**
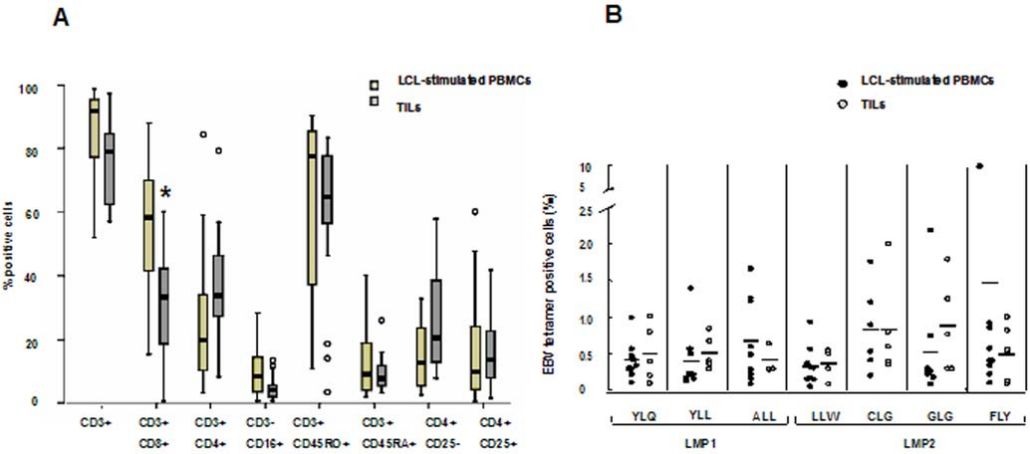
Immunophenotype of LCL stimulated cultures and TILs from NPC patients (A). Surface markers were detected by directly immunofluorescence and FACS analysis in LCL-stimulated cultures (n = 13) and tumor infiltrating lymphocyte (n = 25) expanded for 1 to 4 weeks in IL-2 medium without antigen stimulation. A relative decrease of CD8+ cells and increase of CD4+ cells was observed in TILs compared to LCL-stimulated cultures; both cultures showed high percentage of CD3+CD45RO+ and CD4+CD25+ cells. Frequency of LMP1 and LMP2 epitope-specific T cells in auto-LCL stimulated cultures and TILs from NPC patients (B). LMP1 and LMP2 epitopes specific T cells were detected by EBV tetramer staining and FACS analysis in autologous LCL-stimulated PBMCs (n = 9) and TILs (n = 6) from NPC patients.

In order to investigate whether T cells capable of recognizing the malignant cells are present in the tumor, TILs were isolated from 25 NPC biopsies and then expand in medium containing low doses of IL-2 without additional stimulation until the cell number was sufficient for analysis (Figure 6). Similar to LCL-stimulated cultures the TILs contained a high proportion of cells expressing a CD45RO^+^CD3^+^ memory cell and CD25^+^ activated T cell phenotype while CD45RA^+^CD3^+^ and CD4^+^CD25^−^ naïve T cells and CD3^−^CD16^+^ NK cell were strongly decreased compared to PBMCs (compare Figure 2 and Figure 6A). A relative increase of CD3^+^CD4^+^ cells and decrease of CD3^+^CD8^+^ cells was also observed compared to LCL-stimulated cultures, but the differences were not significant.

The presence of T cells specific for LMP1 and LMP2 epitope was investigated in TILs from 6 HLA-A2 positive patients (Figure 6B). The frequency of T cells stained by the LMP1 and LMP2 tetramers was higher compared to PBMCs in all cases, suggesting that T cells specific for these epitopes are enriched in the tumor. Although the TILs resembled in many respects LCL-activated cultures, comparison of paired cultures revealed a different representation T-cells specific for LMP1 and LMP2 epitopes. While T cells recognizing YLL, ALL and CLG epitopes were preferentially expanded upon LCL-stimulation, T cells specific for the YLQ, LLW and GLG epitopes were preferentially enriched in TILs (Table 4).

**Figure 7:**
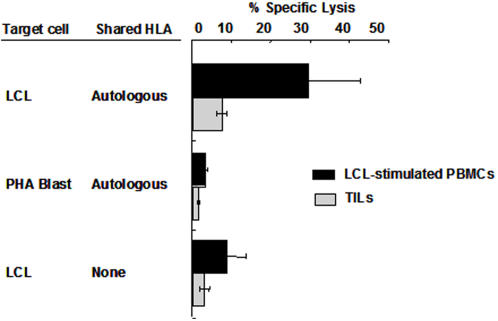
Cytotoxic activity of LCL-stimulated cultures and TILs from NPC patients. The mean ±SD percentage lysis at 10∶1 E∶T ratio of LCL-stimulated cultures (n = 15) and TILs (n = 9) from NPC patients is shown in the figure.

The presence of EBV specific cytotoxic activity was investigated in 10 TIL cultures (Figure 7). The levels of specific lysis against all targets tested were below 10% at 10∶1 E∶T ratio in every cases. The lack of cytotoxic capacity was also confirmed by CD107a staining after stimulated by different targets cells or peptides (Supplementary Information Figure S4 and Table S3). To further investigate whether EBV specific responses could be detected by more sensitive assays, six of the TIL cultures were tested for their capacity to produce IFNγ in response to stimulation with autologous LCLs by EILSPOT assay or intracellular staining (Table 5 and Supplementary Information Table S4). In line with the efficient reactivation of EBV-specific cytotoxicity, high frequencies of EBV-specific T lymphocytes were detected by ELISPOT assays in the PBMCs from patients and controls while the frequency of IFNγ spot forming cells was significantly lower in TILs. Similar results were obtained when IFNγ production was assessed by intracellular staining. Intracellular staining of cells treated with PMA and inomycin confirmed that the TILs were able to produce IFNγ at levels comparable to those observed in LCL-stimulated cultures, whereas the response to autologous LCLs was only marginally different from that induced by stimulation with autologous PHA blast or mismatched LCLs.

**Table 5:** Production of IFNγ by TILs.

	IFN-γ producing cells					
	ELISPOT^a^		(%) Intracellular staining^b^			
	Control	Auto LCL	Control	PMA inomycin	Auto LCL	PHA blast
**P3**	2	2	0.3	32.8	0.7	0.2
**P4**	-	-	0.5	81.2	0.7	0.5
**P6**	1	6	-	-	-	-
**P10**	2	4	-	-	-	-
**P24**	3	46	0.2	69.2	0.5	0.1
**P31**	-	-	0.2	19.8	0.3	0.3
**Patient PBMC (n = 3)**	2	166	-	-	-	-
**Patient ASLC (n = 11)**	-	-	1.2	40.6	5.6	1.7
**Healthy PBMC (n = 3)**	3	230	-	-	-	-
**Healthy ASLC (n = 8)**	-	-	1.2	36	6.1	1.2

a Number of spot-forming cells (SFC)/10^6^.

b Percentage of IFN-γ producing cell detected by intracellular staining.

## Discussion

Conventional treatment for NPC frequently fails and is often accompanied by severe long-term side effects [20]. This, together with the regular association of undifferentiated NPC with EBV infection, and the expression of immunogenic viral antigens in the tumor cells, has motivated strong efforts to develop strategies of immune intervention that could complement or even substitute current therapeutic regimens (reviewed in [21]. The success of this endeavor is tied to a better understanding of the reasons for the failure of endogenous immunity.

Our study performed on a relatively large number of newly diagnosed NPC patients confirms previous observations indicating that the tumor carrier status is associated with an imbalance in the control of EBV infection [22]. The increased antibody titers to structural antigens and the presence of IgA antibodies to VCA and EA and viral DNA in the serum (Figure 1) suggest that productive infection occurs at mucosal sites. This, together with the tumor mass, is likely to cause inflammatory responses that are revealed by the high serum levels of both Th1 and Th2 cytokines (Table 2). Ongoing inflammation may also explain the relative decrease of circulating T lymphocytes and the presence of few T cells expressing activation marker (Figure 3) since activated T cells express adhesion molecules and chemokine receptors that can promote homing to the site of inflammation. The finding that EBV specific CTLs could be reactivated from the blood in the majority of NPC patients (Figure 4) is also in line with previous reports [23], [24] and confirms that overall preserved immunocompetence of the patients. Although EBV specific responses were not detected in approximately one third of the patients (Table 3), this failure did not appear to correlate with the clinical stage of the disease and may due to technical limitation, possibly associated with the use of LCLs carrying a laboratory strain of EBV rather than the endogenous virus. It is noteworthy that although the average age of NPC patients was higher than that of healthy controls, the strongest CTL responses were actually observed in some of the patients. It remains unclear what causes, in the face of persistent T cell response, the impaired control of latent infection reflected by the high antibody titers and presence of EBV DNA in the serum. While viral DNA may be released from the tumor mass due to necrosis, the malignant cells are not a likely source of EBV structural antigens since in the infection is latent in the tumor [25], [26]. Conceivably, EBV reactivation could be triggered by cytokines released at sites of inflammation in the mucosa surrounding the tumor. It remains to be seen whether normal levels T cells specific for EBV lytic antigens are present and functionally competent in the patients.

Our analysis of epitope choice was focused on HLA-A2 restricted epitopes derived from LMP1 and LMP2 since this HLA class I allele was shown to be associated with increased risk to develop NPC in Chinese [27] and, being expressed in the malignant cells, these viral antigens are possible targets of anti-tumor responses. Interestingly, we found that the frequency of T cells capable of recognizing seven different epitopes in these proteins was significantly decreased in the blood of NPC patients compared to healthy donors. The relatively low representation of LMP1 and LMP2 specific T cells was maintained also after stimulation of the PBMCs with autologous LCLs in spite of efficient activation cytotoxic responses, indicating that LMP1 and LMP2 are not dominant targets of EBV specific CTLs NPC patients. This is in line with previous reports showing that, as in healthy EBV carriers, to response of PBMCs from NPC patients to standard LCL-based reactivation protocols is dominated by CTLs directed to epitopes in the nuclear protein EBNA3, 4 and 6 [23], [28]. While the preferential reactivation of EBNA-reactive cells may be driven by the epitope repertoire presented by LCL cells, our finding that T cells recognizing LMP1 and LMP2 epitopes are differently expanded in the patients compared to controls indicates that tumor carriage may be associated a different choice of epitopes. Thus, while is agreement with previous reports [29] two LMP2 epitopes, CLG and FLY, dominated the response in healthy donors, two LMP1 epitopes, YLL and ALL, appeared to be preferentially expanded in NPC patients. This may reflect differences in the functional status of circulating CTL precursors. Although the number of patient is too small to warrant firm conclusions, this observation could have important implications for the choice of antigens and antigen presenting cells in immunotherapy settings.

Analyzing EBV-specific responses in NPC tumor biopsy material, we found that TILs expanded for short time in IL-2 in the absence of exogenous stimuli were unable to lyse autologous EBV infected targets and only a minor proportion of the cells produced IFN-γ upon stimulation with the autologous LCL although the majority could be triggered to do so upon stimulation with PMA and ionomycine. We purposely avoided the use of restimulation protocols and performed our analysis soon after isolation in order to preserve, within the limits of what is feasible given the very small amount of starting material, the characteristics of the original TIL population. It is noteworthy that, although functionally inactive, the expanded TILs expressed an activated memory T cell phenotype similar to that of LCL-stimulated PBMCs and were enriched in T cells specific for all the LMP1 and LMP2 epitopes tested. Thus, the relative decrease of LMP1 and LMP2 specific cells observed in the periphery does not appear to reflect a selective loss of these cells but rather their preferential homing to the tumor. Previous work on NPC biopsy demonstrated that CTL precursors specific for various EBNAs can be reactivated upon stimulation of TILs with autologous LCLs but did not detect any responses to LMP2 or any other NPC-associated viral protein [23]. This was taken to suggest that LMP2-specific responses may be excluded from the tumor site. Our findings offer another explanation, namely that, although appropriate CTL precursors reach the tumor, they are selectively rendered nonfunctional at this location.

Work on tumor biopsies from HD patients have earlier suggested that EBV-specific CTL responses may be inhibited in the EBV positive tumors [18]. Similar to NPC, the malignant Reed-Sternberg cells of HD express EBV latency II and it is therefore conceivable that one or more of the viral proteins that characterize this latency program may be involved in the local suppression of EBV-specific immunity. LMP1 is a prime candidate due to its capacity to induce constitutive activation of NF-kB and the production of cytokines such as IL-10 that is known to suppress cellular immune responses [30], [31], [32], [33], [34], [35]. In line with this possibility we have found significant amount of IL-10 in the serum of 20 out of 27 NPC patients (Table 2). LMP1 may also have a more direct effect on the regulation of EBV specific responses since two LMP1 peptides with strong homology to an immunosuppressive peptide found in the retrovirus-encoded transmembrane protein p15E were shown to strongly inhibit CTL and natural killer (NK) cell activity *in vitro* [36]. It is noteworthy that the TIL cultures were enriched in CD4^+^ cells and a significant proportion of these cells also expressed CD25. CD4^+^CD25^+^ cells expressing the forkhead box (Fox)p3 transcription factor, also known as Treg, are a suppressor T cell population that regulates a variety of immune responses. Recent studies demonstrate that elevated proportions of Treg cells are present in various types of cancers and suppress anti-tumor immunity [37], [38]. Importantly, tumor-specific Treg cells were shown to inhibit immune responses only when exposed to antigens presented by tumor cells [39], [40], [41]. It remains to be seen whether the LMP1 or other viral proteins expressed in NPC may be directly responsible for the activation of T-cells with the capacity to suppress virus-specific immune responses at the tumor site.

Our findings have interesting implications for current efforts to develop therapeutic strategies for NPC based on selective boosting of endogenous immunity or adoptive transfer of CTLs. In spite of a clear imbalance in the control of EBV infection, the majority of NPC patients are capable of mounting efficient responses to LCLs *in vitro.* Hence, boosting these responses could be of therapeutic benefit. However, the different antigen choice observed in NPC patients and the finding that EBV-specific T cells may be inactivated in the tumor, possibly due to exposure to an antigenic repertoire that triggers Treg responses, suggest that the outcome of immunization strategies will be strongly dependent on the choice of antigens and antigen presenting cells. Most importantly, a choice based on the specificity and strength of the responses induced by LCL stimulation is probably not optimal since the tumor cells may present a different set of antigenic peptides and some of the presented epitopes may suppress rather than enhance the anti-tumor effect. Adoptive transfer of CTLs may hold a better chance of success since studies of NPC cell lines indicate that the malignant cells are capable of processing endogenously antigens and at least some of the epitopes are recognized by HLA class I-restricted CTL activated by the currently used reactivation protocols [23], [24], [42]. Our finding that LMP1 and LMP2 specific T cells are enriched in TILs suggests that *in vitro* activated CTLs may also traffic to the tumor but it remains to be determined whether and for how long these cells will remain functional at the tumor site. Thus, the development of means to control the activity of Tregs should become an important focus in the establishment of effective strategies of immune intervention in NPC and, by inference, other EBV associated malignancies expressing latency II.

## Materials and Methods

### Sample collection

Heparinized venous blood and tumor biopsies were collected from 40 untreated primary nasopharyngeal carcinoma (NPC) patients at the time of first remittal to the Nasopharyngeal Carcinoma Unit of the Cancer Center of Sun-yat Sen University. Twenty healthy donors were students and coworkers of the Cancer Center, All patients and healthy donors were South Chinese living in Guangdong province for 2–3 generation. The Ethical Committee of the Sun-yat Sen University Cancer Center approved the study and full consent was obtained from all patients and healthy donors. The NPC histological type and clinical stage were determined according to the UICC/AJCC 1997 classification [43]. A summary of the clinic information, HLA type and EBV status of the patients and controls is shown in Table 1.

### Study design

Peripheral blood mononuclear cells (PBMCs) were isolated from 30 ml heparinized blood, by Ficoll/Isopaque gradient fractionation and monocytes were depleted by plastic adherence. 10^7^ PBMCs were used for EBV infection, PHA activation, phenotype analysis and EBV tetramer staining. The remaining cells were cryopreserved for activation of EBV specific CTLs once the autologous LCL became available. Serum samples were collected for determination of EBV specific antibodies, EBV DNA load, and cytokines levels. The tumor biopsies were divided into small portions for histopathology investigation and, when possible, analysis of EBV status by EBER1 in situ hybridization. When available, one portion was used for isolation of tumor infiltrating lymphocytes and the remaining portion was used to set up primary tumor cell cultures.

### In situ hybridization (ISH)

Paraffin embedded NPC sections were subjected to antigen retrieval by dewaxing for 15 min at 95°C in 0.01 M sodium citrate buffer in a steam cooker. EBER expression was detected by ISH using the Epstein-Barr Virus Probe ISH Kit (Novocastra Laboratories Ltd, Newcastle, UK) according to the instruction of the manufacturer.

### Serum EBV antibody titers, DNA load and cytokine levels

IgG and IgA antibody titers against the EBV virus capsid antigen (VCA) and early antigen (EA) were detected using a commercial enzyme-labeling immunostaining assay detection kit (Zhongshan Biotechnology Company, Tianjin, China) according to the instruction of manufacturer. EBV DNA load was determined by real time quantitative PCR in DNA extracted from 250 µl serum with the QIAamp Blood Kit (Qiagen, Hilden, Germany) using the probe W-67T derived from the BamHI-W region of the EBV genome. The procedures for real-time quantitative PCR and reaction set-up were carried out as described previously [44]. Fifty µl of serum were analyzed for cytokine content, using the human Th1/Th2 cytokine Cytometric Bead Array Kit (BD-Bioscience/Pharmingen, San Diego, CA) as described [45]. Fluorescence was detected using a FACS Aria^TM^ and the data were analyzed using the CBA software (BD Bioscience).

### Isolation of tumor infiltrating lymphocytes (TILs) and culture of primary tumor cells

The NPC biopsies were minced and digested with 100 µg/ml Collagenase IV (Sigma, St. Louis, USA) for 2–3 h at 37°C. TILs were isolated from the single cell suspension by Ficoll-Hypaque gradient centrifugation (Zhongshan biocompany, Tianjin, China), and 2×10^6^ cells were cultured for 1 to 4 weeks in RPMI 1640 supplemented with antibiotics, 10% fetal calf serum complete (FCS, GIBO), and 20 IU/ml IL-2 (R&D Systems Inc, Minneapolis, USA) in 24-well tissue culture plates. When available, a small aliquot of the NPC tissues was finely minced and the fragments were placed in a 25 cm^2^ tissue culture flask coated with type I collagen (Sigma). The adherent cells were maintained in Keratinocyte-SFC medium supplemented with 20 mM L-glutamine, 5 ng/ml human recombinant epidermal growth factor and 50 mg/ml bovine pituitary extract (Invitrogen). The NPC primary culture cells were expansion in vitro for 5 to 8 generations and then frozen for later use. The phenotype of the NPC primary culture cells is shown in Supplementary Information Figure S1.

### Flow cytometry and tetramer staining

PerCP, FITC or PE-conjugated mouse monoclonal antibodies (MAbs) to CD3 (Clone UCHT1), CD4 (Clone RPA-T4), CD8 (Clone RPA-T8), CD16 (Clone 3G8), CD45RA (Clone HI100), (Clone UCHL1) from BD Pharmingen Company (San Jose, CA, USA) and FITC-conjugated anti-human CD4 (Clone RPA-T4) and PE-conjugated anti-human CD25 (BC96) from eBioscience (San Diego, CA, USA) were used for surface markers analysis. The cells were incubated with appropriate fluorochrome conjugated MAb at 4°C for 30 min, followed by three washes in phosphate-buffered saline (PBS). Fluorescence was detected with a FACS Aria^TM^ and data was analyzed with FACS Diva software (BD Biosciences).

The frequency of T cell specific for HLA-A2 restricted epitopes was analyzed by staining with HLA-A2 tetramers assembled with synthetic peptides from LMP1: YLQQNWWTL, YLLEMLWRL, ALLVLYSFA and LMP2: CLGGLLTMV, FLYALALLL, GLGTLGAAI, LLW TLVVLL (Guangzhou Taimo Corporation, Guangzhou, China). Aliquots of 0.5–1×10^6^ cells were incubated at 4°C for 30 minutes in PBS containing 1% FCS and 1 µg of phycoerythrin (PE)–labeled tetramers. The samples were co-stained with anti-CD8-FITC and anti-CD3-PerCP MAbs followed by fixation in 0.5% paraformaldehyde [46]. At least 10^5^ cells per sample were analyzed.

### Generation of EBV transformed LCLs and CTL cultures

LCLs were generated by EBV infection of 2×10^6^ PBMCs with spent supernatant from the B95-8 virus producing cell line [47], in the presence of 1 mg/ml cyclosporin A (Sigma). Cryopreserved PBMCs were thawed and aliquots of 3×10^6^ cells were stimulated in 24 well plates with irradiated (4000 Rads) LCL cells at 40∶1 responder:stimulator (R∶S) ratio. After 7 days, viable cells were re-stimulated under the same conditions and, 3 days later, the cells were expanded in complete medium containing 10 UI/ml IL-2 (R&D Systems Inc). The cultures were restimulated weekly. For generation of PHA blasts PBMCs were stimulated with 1 µg/ml PHA-L (Sigma) for 3 days and expanded in IL-2 containing medium. The HLA type was determined by sequence specific primer (SSP)-PCR in the Guangzhou Organ Transplant and Tissue Typing Center.

### Cytotoxicity assay

Cytotoxic activity was evaluated in standard 4h ^51^Cr release assays, as previously described [48]. The targets panel included autologous LCLs and PHA blasts, allogeneic HLA class I mismatched or, when available, single HLA class I allele matched LCLs and autologous primary NPC cells. All assays were run in triplicate and the results are presented as the mean±SD.

### Intracellular cytokine and CD107a staining

Five ×10^5^ cells were stimulated with PMA/inomycin (0.5–1 mg/ml), auto-LCL at 3∶1 R∶S ratio or EBV peptide (20 µg/ml) in 200 µl of complete RPMI 1640 medium in 96-well round tissue culture plate, in the presence of PE-conjugated mouse anti-human CD107a antibody(Clone H4A3 ) and GolgiPlug (BD Phar-Mingen, San Diego, CA) for 4 h at 37°C in dark. Intracellular cytokine staining was performed using the Cytofix/Cytoperm Plus kit (BD PharMingen).

### ELISPOT assay

ELISPOT assays were performed as previously described [49]. Five ×10^5^ cells were plated on Multi-Screen Immobilon-P filtration plates (Millipore, Bedford, MA) pre-coated with IFN-γ-specific mAbs (Ucytech Biosciences, Netherlands) and then incubated overnight with the auto-LCL, HLA class I matched LCL at 10∶1 R∶S, or 20 µg/ml of synthetic EBV peptides. The IFN-γ producing cells were detected as red spots by using a human IFNγ ELISPOT kit (U-CyTech Biosciences Company, Netherlands) and counted with the ImmunospotTM Acessories software (Cellular Technology Ltd, Carnegie Avenue, Cleveland, USA). All assays were performed in duplicate. Spontaneous IFNγ production was detected by omitting the stimulation and maximal activity was measured by stimulation with 10 µg/ml PHA.

### Statistical analysis

Statistical analysis was performed with the SSPS 13.0 software package (SPSS Inc, Chicago, USA), using Chi-square test, *t* student test, or Mann-Whitney U test, with level of significance set at P<0.05.

## Supporting Information

Table S1 (0.06 MB DOC)

Table S2 (0.08 MB DOC)

Table S3 (3.18 MB DOC)

Table S4 (0.03 MB DOC)

Figure S1 Characteristics of NPC primary culture cells. Morphology of NPC primary culture cells under light microscope (A and B). Keratin staining (C). EBERs in situ hybridization in NPC primary cells (C). Negative control for EBERs staining in normal nasopharyngeal epithelia (D)
(3.45 MB DOC)

Figure S2 The frequency of T cells specific for HLA-A2 restriction epitopes in EBV LMP1 and LMP2 in HLA-A2 positive NPC patient and healthy donor. The frequencies of tetramer positive cells in CD3+CD8high PBMCs and TILs from NPC patient P24, and in PBMCs and auto-LCL stimulated PBMCs from healthy donor N16 are shown in this figure.
(3.27 MB TIF)

Figure S3 Cytotoxicity analysis of auto-LCL stimulated PBMCs from NPC patient 1. Polyclonal CTL cultures were tested for cytotoxic activity against autologous PHA blasts loaded with LMP1 or LMP2 peptides in 4 hrs 51Cr release assays(A); or stimulated with LMP1 or LMP2 peptides for 4 hrs in round 96-well tissue culture plate followed by intracellular staining for CD107a and IFN-γ(B).
(3.18 MB TIF)

Figure S4 Cytotoxicity analysis of TILs from NPC patient 31. Tumor infiltrating lymphocyte (P31) expanded for 1 to 4 weeks in IL-2 medium without antigen stimulation, were tested for cytotoxic activity against autologous PHA blasts loaded with LMP1 or LMP2 peptides in 4 hrs 51Cr release assays (A); or co-cultured with different targets cells (E∶T = 10∶1) for 4 hrs in round 96-well tissue culture plate followed by intracellular staining for CD107a and IFN-γ(B).
(3.22 MB TIF)
