# Supplementary material for: Functional Inactivation of EBV-Specific T-Lymphocytes in Nasopharyngeal Carcinoma: Implications for Tumor Immunotherapy
Source: PLoS One. 2007 Nov 7;2(11):e1122. doi: 10.1371/journal.pone.0001122 (PMC2048575; doi:10.1371/journal.pone.0001122)
Supplement: Table S1 — (0.06 MB DOC) [file pone.0001122.s001.doc]

**Table S1. Percentage of CD3^+^CD107a^+^** cell in auto-LCL stimulated cultures from NPC patients and healthy donors

| Sample | %CD3^+^CD107a^+^ | | | | | | | | | | |
| --- | --- | --- | --- | --- | --- | --- | --- | --- | --- | --- | --- |
|  | Medium | PMA/ionocymin | Auto-LCL | Auto-PHA | YLQ | YLL | ALL | GLG | FLY | LLW | CLG |
| P1 | 10.5 | 16.7 | - ^a^ | - | 11.8 | 8.5 | 7.1 | 7 | 8.2 | 7.5 | 6.9 |
| P13 | - | - | - | - | 3.2 | 3.6 | 2.3 | 3.1 | 3.4 | 3.2 | 2.8 |
| P17 | 6.3 | 32.2 | - | - | 6.9 | 8.1 | 7.5 | 8.2 | 6.7 | 7.9 | 6.9 |
| P30 | 2.9 | 14.1 | 13.5 | 3.4 | 11.6 | 11 | 3.5 | 2.9 | 2 | 2.8 | 2 |
| P31 | 1.7 | 18.4 | 9 | 1.7 | 2.1 | 2 | 2.3 | 2.3 | 2.1 | 1.9 | 1.9 |
| P34 | 4.8 | 18.5 | 7.8 | 2.7 | - | - | - | - | - | - | - |
| P37 | 1.5 | 4.4 | 5.4 | 1.5 | - | - | - | - | - | - | - |
| P40 | 0.8 | 1.4 | 1.4 | 1 | - | - | - | - | - | - | - |
| Mean | 4.1 | 15.1 | 7.4 | 2.1 | 7.1 | 6.6 | 4.5 | 4.7 | 4.5 | 4.7 | 4.1 |
| N20 | 1.6 | 53.9 | 16.3 | 3.4 | 3.6 | 3.9 | 1.9 | 2.6 | 1.6 | 2.5 | 1.9 |
| N6 | 2.9 | 20.7 | 5.1 | 2.6 | 2.9 | 8 | 2.5 | 4.1 | 3 | 2.8 | 3.7 |
| N5 | - | - | - | - | 2.7 | 2.5 | 2.4 | 4.3 | 25.8 | 2.3 | 3.6 |
| Mean | 2.3 | 37.3 | 10.7 | 3.0 | 3.1 | 4.8 | 2.3 | 3.7 | 10.1 | 2.5 | 3.1 |

a. - = not done
