# Supplementary material for: Functional Inactivation of EBV-Specific T-Lymphocytes in Nasopharyngeal Carcinoma: Implications for Tumor Immunotherapy
Source: PLoS One. 2007 Nov 7;2(11):e1122. doi: 10.1371/journal.pone.0001122 (PMC2048575; doi:10.1371/journal.pone.0001122)
Supplement: Table S2 — (0.08 MB DOC) [file pone.0001122.s002.doc]

**Table S2. The percentage of IFN-γ positive cell in LCL-stimulated PBMCs from NPC patients and healthy controls**

| Sample | % IFN-γ producing Cell^a^ | | | | | | | | | | |
| --- | --- | --- | --- | --- | --- | --- | --- | --- | --- | --- | --- |
|  | Medium | PMA/ionocymin | Auto-LCL | Auto-Blast | YLQ | YLL | ALL | GLG | FLY | LLW | CLG |
| P1 | 0.8 | 28.1 | 5.6 | 1.9 | 2.4 | 2.5 | 1.7 | 2.1 | 1.9 | 2.1 | 2.1 |
| P5 | 2.7 | 33 | 11.3 | 2.6 | -^a^ | - | - | - | - | - | - |
| P13 | 0.8 | 43.5 | 4.3 | 0.5 | 1.2 | - | - | - | - | - | 0.8 |
| P17 | 0.4 | 85.7 | 14 | 0.7 | 0.4 | - | - | - | - | - | 0.5 |
| P19 | 0.2 | 46.4 | 3.8 | 0.8 | - | - | - | - | - | - | - |
| P20 | 1.5 | 89.7 | 6 | 1.8 | 1.2 | - | - | - | - | - | 0.7 |
| P21 | 3.6 | 37 | 4.1 | 1.4 | 0.7 | - | - | - | - | - | 1.3 |
| P30 | 0.6 | - | 8.2 | 0.5 | 1 | 0.5 | 0.7 | 0.6 | 0.4 | 0.7 | 0.7 |
| P31 | 0.3 | 21 | 5.5 | 0.6 | 0.7 | 0.5 | 0.7 | 0.9 | 0.5 | 0.7 | 0.7 |
| P34 | 1.8 | 25.5 | 2.1 | 0.8 | - | - | - | - | - | - | - |
| P37 | 0.1 | 2.6 | 2.8 | 0.2 | - | - | - | - | - | - | - |
| P40 | 0.1 | 2.2 | 1.4 | 0.2 | - | - | - | - | - | - | - |
| Mean | 1.1 | 37.7 | 5.6 | 1.0 | 1.1 | 1.2 | 1.0 | 1.2 | 0.9 | 1.2 | 1.0 |
| N5 | 0.8 | 27.2 | 6.6 | 0.2 | 0.7 | - | - | - | - | - | 0.8 |
| N6 | 2.1 | 38.2 | 3.5 | 1.8 | 2.7 | 1.7 | 1.8 | 2.2 | 2.1 | 2.3 | 2 |
| N14 | 0.4 | 40.5 | 5.5 | 0.1 | 0.1 | 0.1 | 0.1 | 0.2 | 0.2 | 0.4 | 0.1 |
| N16 | 0.8 | 3.7 | 5.5 | 0.9 | 1.7 | 0.7 | 0.7 | 1 | 1 | 2.2 | 1.9 |
| N12 | 1.7 | 61.2 | 4.6 | 1.2 | - | - | - | - | - | - | - |
| N15 | 0.3 | 15.4 | 4 | 0.3 | - | - | - | - | - | - | - |
| N18 | 2.2 | 50.2 | 4.1 | 2.6 | - | - | - | - | - | - | - |
| N20 | 0.6 | 55.7 | 14.7 | 3 | 0.7 | 0.6 | 0.7 | 0.5 | 0.9 | 0.5 | 0.7 |
| Mean | 1.1 | 36.5 | 6.1 | 1.3 | 1.2 | 0.8 | 0.8 | 1.0 | 1.1 | 1.4 | 1.1 |

1. “-“ = not done
