# Supplementary figures and images for: Functional Inactivation of EBV-Specific T-Lymphocytes in Nasopharyngeal Carcinoma: Implications for Tumor Immunotherapy
Source: PLoS One. 2007 Nov 7;2(11):e1122. doi: 10.1371/journal.pone.0001122 (PMC2048575; doi:10.1371/journal.pone.0001122)

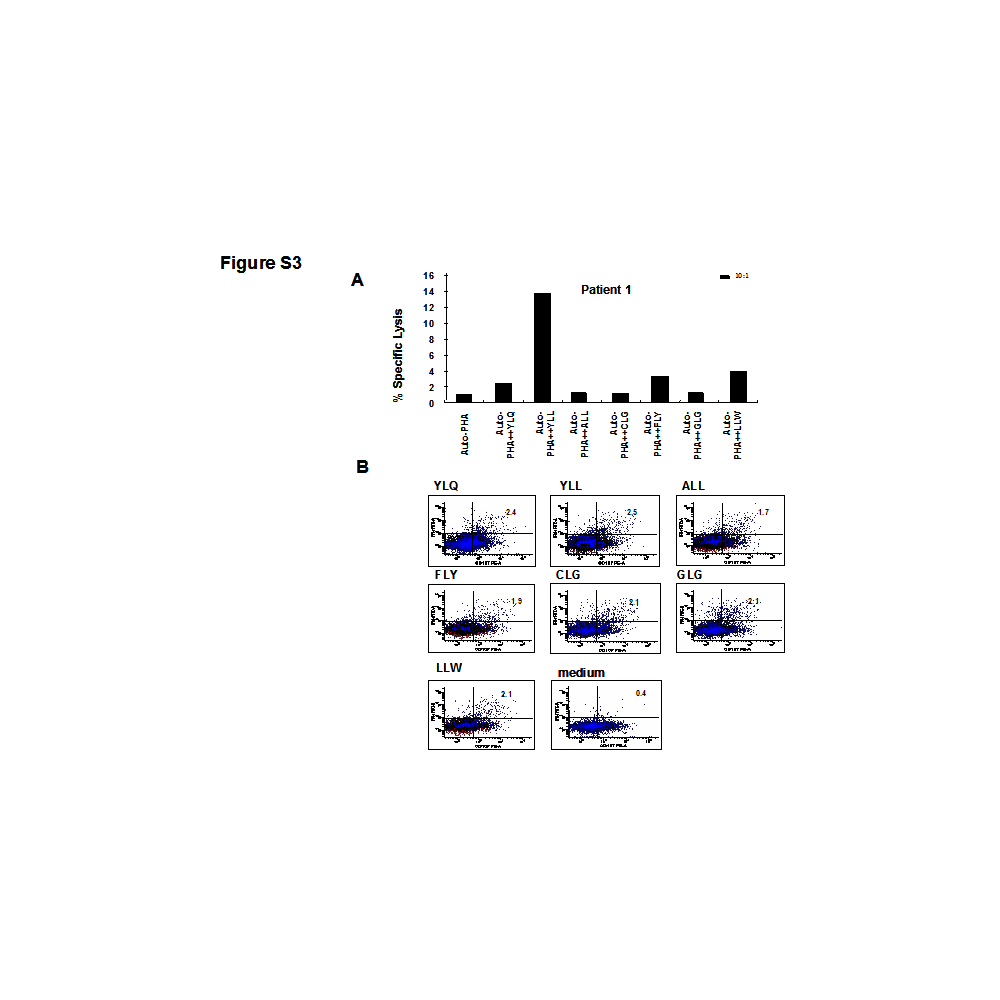

Supplement: Table S3 — (3.18 MB DOC) [file pone.0001122.s003.doc]

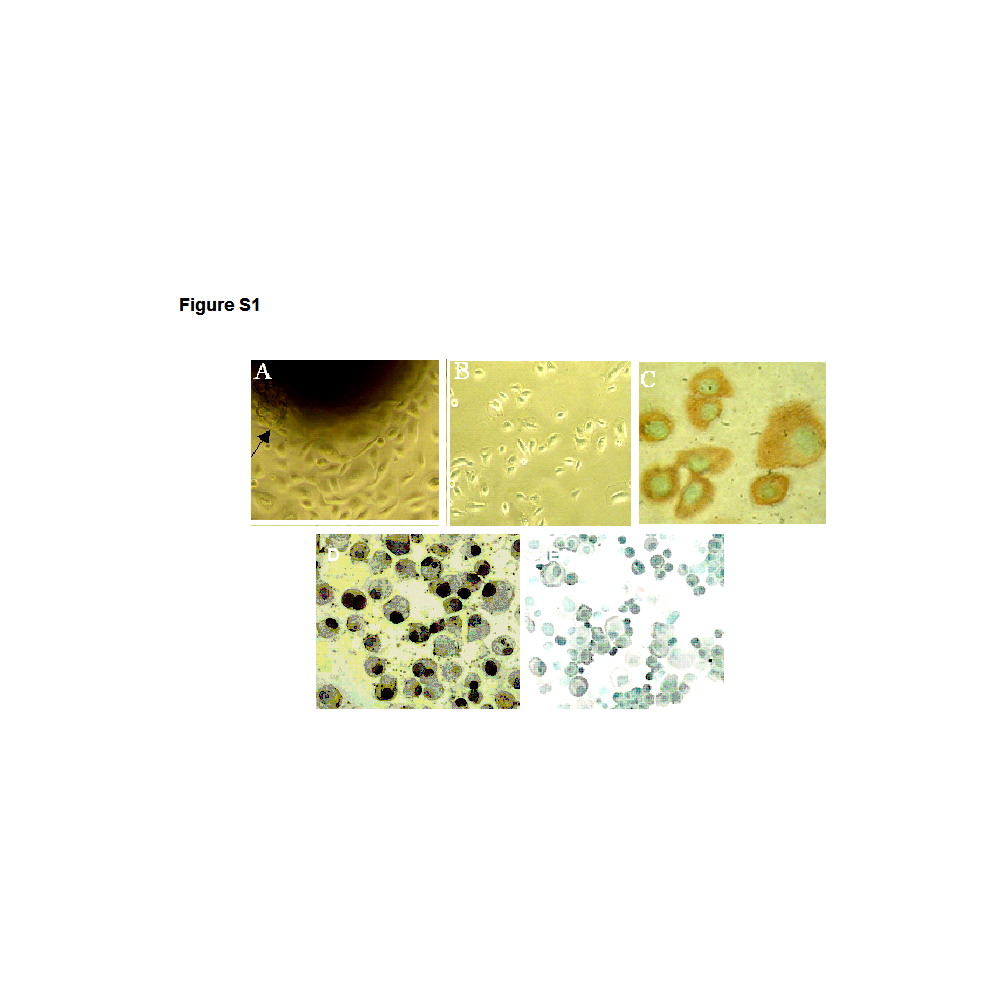

Supplement: Figure S1 — Characteristics of NPC primary culture cells. Morphology of NPC primary culture cells under light microscope (A and B). Keratin staining (C). EBERs in situ hybridization in NPC primary cells (C). Negative control for EBERs staining in normal nasopharyngeal epithelia (D) (3.45 MB DOC) [file pone.0001122.s005.doc]

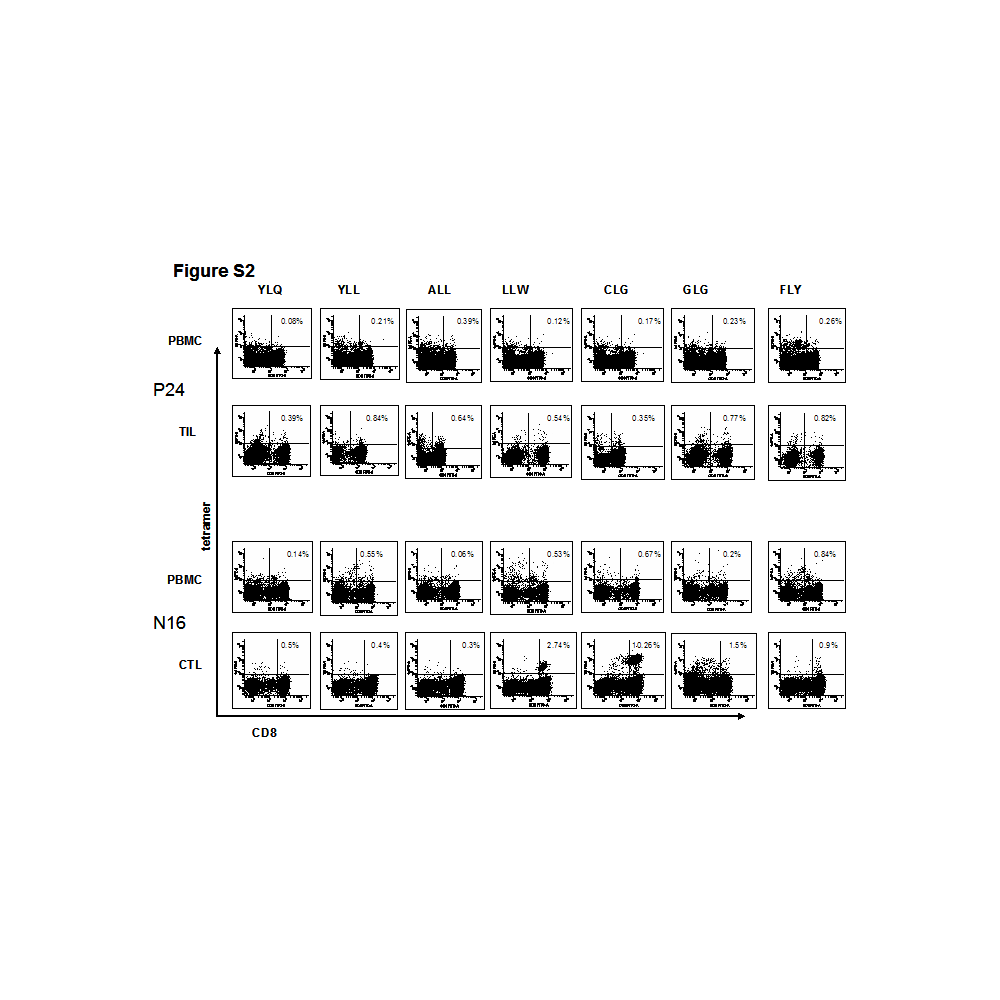

Supplement: Figure S2 — The frequency of T cells specific for HLA-A2 restriction epitopes in EBV LMP1 and LMP2 in HLA-A2 positive NPC patient and healthy donor. The frequencies of tetramer positive cells in CD3+CD8high PBMCs and TILs from NPC patient P24, and in PBMCs and auto-LCL stimulated PBMCs from healthy donor N16 are shown in this figure. (3.27 MB TIF) [file pone.0001122.s006.tif]

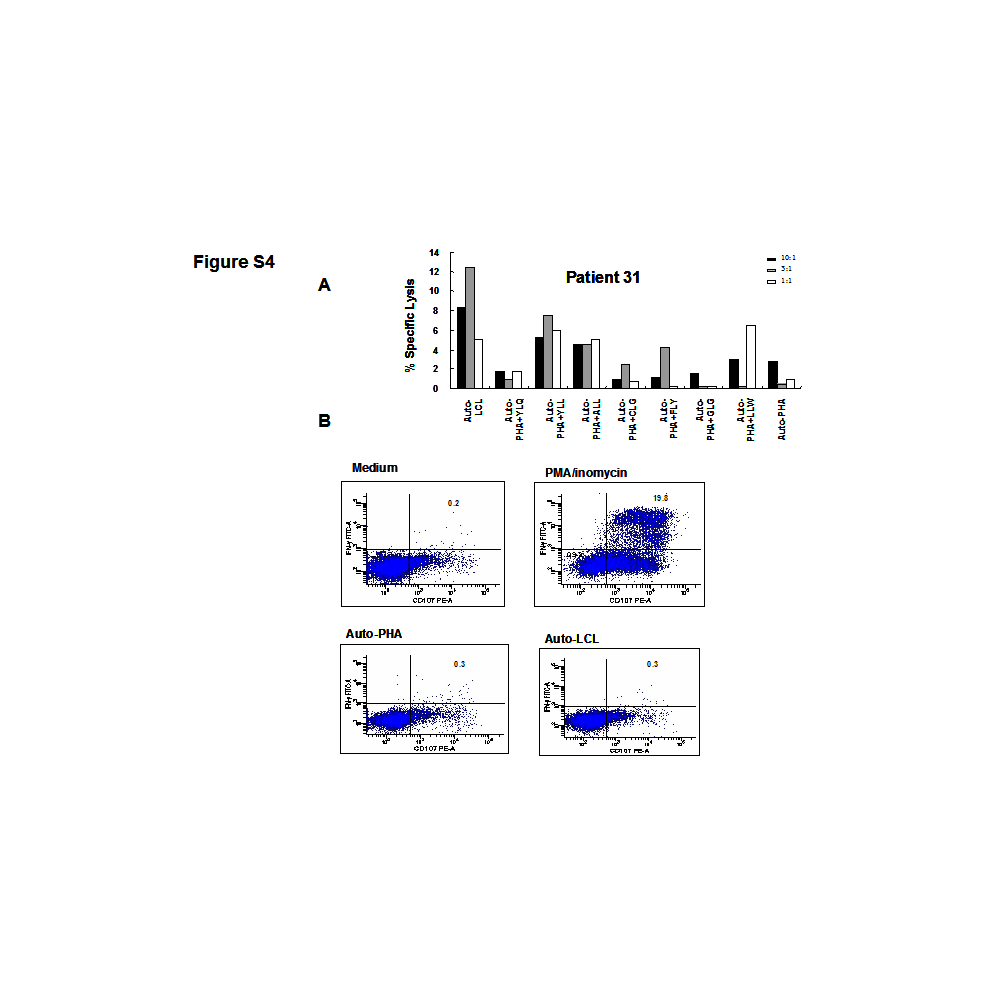

Supplement: Figure S4 — Cytotoxicity analysis of TILs from NPC patient 31. Tumor infiltrating lymphocyte (P31) expanded for 1 to 4 weeks in IL-2 medium without antigen stimulation, were tested for cytotoxic activity against autologous PHA blasts loaded with LMP1 or LMP2 peptides in 4 hrs 51Cr release assays (A); or co-cultured with different targets cells (E∶T = 10∶1) for 4 hrs in round 96-well tissue culture plate followed by intracellular staining for CD107a and IFN-γ(B). (3.22 MB TIF) [file pone.0001122.s008.tif]
