# Supplementary material for: Functional Inactivation of EBV-Specific T-Lymphocytes in Nasopharyngeal Carcinoma: Implications for Tumor Immunotherapy
Source: PLoS One. 2007 Nov 7;2(11):e1122. doi: 10.1371/journal.pone.0001122 (PMC2048575; doi:10.1371/journal.pone.0001122)
Supplement: Table S4 — (0.03 MB DOC) [file pone.0001122.s004.doc]

**Table S4. Percentage of IFN-γ positive cells in TILs after the stimulation with LMP1 and LMP2 peptides**

| Sample | % IFN-γ producing Cell | | | | | | | | | | |
| --- | --- | --- | --- | --- | --- | --- | --- | --- | --- | --- | --- |
|  | Medium | PMA/ionocymin | Auto-LCL | Auto-Blast | YLQ | YLL | ALL | GLG | FLY | LLW | CLG |
| P3 | 0.3 | 32.8 | 0.7 | 0.2 | -^a^ | - | - | - | - | - | - |
| P4 | 0.5 | 81.2 | 0.7 | 0.5 | - | - | - | - | - | - | - |
| P24 | 0.2 | 69.2 | 0.5 | 0.1 | 0.1 | 0.3 | 0.2 | 0.2 | 0.1 | 0.3 | 0.1 |
| P31 | 0.2 | 19.8 | 0.3 | 0.3 | - | - | - | - | - | - | - |

a. - = not done
